# The Challenge of Choosing the Right Stimulation Target for Dystonic Tremor—A Series of Instructive Cases

**DOI:** 10.1002/mdc3.13846

**Published:** 2023-08-22

**Authors:** Steffen Paschen, Jos S. Becktepe, Markus A. Hobert, Kirsten E. Zeuner, Ann‐Kristin Helmers, Daniela Berg, Günther Deuschl

**Affiliations:** ^1^ Department of Neurology University Hospital Schleswig‐Holstein, Campus Kiel and Christian Albrechts‐University of Kiel Kiel Germany; ^2^ Department of Neurosurgery University Hospital Schleswig‐Holstein, Campus Kiel and Christian Albrechts‐University of Kiel Kiel Germany

**Keywords:** dystonic tremor, dystonia, thalamic/pallidal deep brain stimulation

## Abstract

**Background:**

Thalamic deep brain stimulation (DBS) is established for medically refractory tremor syndromes and globus pallidus stimulation (GPi‐DBS) for medically refractory dystonia syndromes. For combined tremor and dystonia syndromes, the best target is unclear.

**Objectives:**

We present four patients with two different profiles whose clinical course demonstrates that our current analysis of clinical symptomatology is not a sufficient predictor of surgical success.

**Methods:**

Outcome parameters were assessed with observer‐blinded video ratings and included the Fahn‐Tolosa‐Marin‐Tremor Rating Scale (FTM‐TRS) and the Unified Dystonia Rating Scale (UDRS).

**Results:**

Two patients with “predominant lateralized action tremor” of the hands and mild cervical dystonia showed no relevant tremor improvement after GPi‐DBS, but UDRS improved (mean, 45%). Rescue ventral intermediate nucleus of the thalamus (Vim)‐DBS electrodes were implanted and both patients benefited significantly with a mean tremor reduction of 51%.

Two other patients with “axial‐predominant action tremor of the trunk and head” associated with cervical dystonia underwent bilateral Vim‐DBS implantation with little effect on tremor (24% reduction in mean FTM‐TRS total score) and no effect on dystonic symptoms. GPi rescue DBS was implanted and showed a significant effect on tremor (63% reduction in mean FTM‐TRS) and dystonia (49% reduction in UDRS).

**Conclusions:**

The diagnosis of dystonic tremor alone is not a sufficient predictor to establish the differential indication of GPi‐ or Vim‐DBS. Further criteria (eg, proximal‐distal distribution of tremor/dystonia) are needed to avoid rescue surgery in the future. On the other hand, the course of our patients encourages rescue surgery in such severely disabled patients if the first target fails.

For patients with medically refractory disabling essential tremor (ET) or dystonia, deep brain stimulation (DBS) is well established and recommended.^1^, ^2^ In ET patients, the standard targets are the ventral intermediate nucleus of the thalamus (Vim) and the posterior subthalamic area (PSA),^3^, ^4^ both of which likely hit the dentato‐rubro‐thalamic tract in different segments influencing the cerebello‐thalamo‐cortical network.^5^, ^6^ In dystonia, high frequency stimulation of the internal pallidum (GPi) has been shown to have a good effect on involuntary muscle activity.^7^, ^8^ Less frequent, the subthalamic nucleus (STN) is used as a target for treating dystonia.^9^

In combined syndromes of tremor and dystonia, both symptoms occur. These encompass ET plus dystonic soft signs, dystonic tremor (DT), and dystonia associated with tremor.^10^ Target selection for patients with concomitant tremor and dystonic symptoms has not yet been firmly established. If the Vim is chosen, dystonic symptoms are improved only to a small extent, if at all,^11^ and if GPi is chosen, severe tremors may not ameliorate enough to achieve functional gains in activities such as eating, drinking, and writing. For patients with an inadequate response to one stimulation site, “rescue lead” implantation targeting the second stimulation site has been proposed.^12^

In addition, other factors such as the anatomic distribution of the tremor (ie, midline/axial versus distal/arm tremor) may influence the choice of target.^13^

Here, we present four instructive cases with severe action tremor syndromes combined with idiopathic cervical dystonia: two patients with “predominant lateralized action tremor of the hands and mild cervical dystonia” and two patients with an unusual, rapidly progressive “axial‐predominant action tremor of the trunk and head” combined with cervical dystonia and dystonic hand posturing. In all four cases, the tremor was the main disabling symptom, whereas the dystonia was only of mild severity. All patients underwent an initial bilateral DBS implantation of Vim or GPi, which was unsuccessful. Therefore, all patients underwent a second surgery with bilateral implantation of the other target (GPi or Vim). Therefore, four electrodes (bilateral Vim and bilateral GPi) were consecutively implanted in each patient.

## Methods

In this case series, we included four patients with severe action tremor syndromes combined with dystonia who had undergone staged DBS surgery of both the Vim and the GPi in the Department of Neurology/Neurosurgery of the University Hospital Schleswig‐Holstein, Kiel Campus at different times during the past 8 years. Data on demographics, disease course, medical treatment, DBS surgery, and follow up were extracted. Tremor and dystonia severity were assessed by blinded video rating (S.P., J.B.) using the Fahn‐Tolosa‐Marin Tremor Rating Scale (FTM‐TRS)^14^ and the Unified Dystonia Rating Scale (UDRS).^15^

All assessments at baseline and follow‐up were video recorded. Follow‐up examinations were performed 3 to 8 months after implantation of the first and second pair of electrodes (into Vim or GPi, respectively). The DBS operations and setting of stimulation parameters were done according to the local established standard as described previously.^16^, ^17^ In all patients, the correct electrode position were verified postoperatively by computed tomography. Outside the formal assessments, the patients were seen on an outpatient‐basis to optimize stimulation 2 to 7 times each patient.

Publication of this case series was approved by the local ethics committee (D601/20). All patients gave their written consent for video documentation and publication.

### Statistical Analysis

Based on the blinded video rating, the mean FTM‐TRS (parts A–C and total score) and UDRS of both raters were calculated for cases 1 and 2 and for cases 3 and 4, respectively. Interrater reliability was determined using Cohen's unweighted κ. Statistical analysis was performed using JASP Team (version 0.16, University of Amsterdam, Netherlands). Blinded video rating showed high interrater reliability (Cohen's unweighted κ: UDRS 0.9; TETRAS: 0.8).

## Results

The four patients were between 66 and 72 years old at the time of the first DBS surgery (Table 1). According to the clinical syndrome, they were divided into two groups.

**TABLE 1:** Baseline data

	Case 1	Case 2	Case 3	Case 4
Age (years) at first DBS implantation, gender	73, m	66, m	70, f	71, f
Disease duration until first DBS operation (years)	28	23	5	9
Family history of tremor	Negative	Mother with Parkinson's disease	Negative	Brother and sister with tremor syndrome
Alcohol response (yes/no/unclear)	Yes	Yes	Unclear	No
Tremor main manifestation site	Arm (right>left, action tremor)	Arm (right>left, action tremor)	Trunk (postural axial tremor)	Trunk (postural axial tremor)
Dystonia manifestation	Laterocollis to the right, torticollis to the left	Laterocaput to the left, torticollis to the right, left shoulder elevation, reduced arm swing right, wrist flexion right, first manifestation with writer's cramp of the right arm.	Mild torticollis to the right, laterocollis to the left	Retrocaput, anterior shift, laterocaput to the left, dystonic posturing of both hands, shoulder elevation left, sensory trick
EMG peak frequency (Hz)	5	5	5	4
FTM‐TRS total score at baseline (points)	67	68	105	58
Anti‐tremor medication	Primidone, metoprolol, topiramate	Primidone, propranolol, topiramate, levetircacetam, trihexyphendyl, levodopa, botulinumtoxine	Primidone	Primidone, propranole, topiramate, botulinumtoxine, bromazepam
First DBS target	GPi	GPi	Vim	Vim
Time between first and second DBS implantation (months)	11	7	12	5

Abbreviations: DBS, deep brain stimulation; m, male; f, female; EMG, electromyography; FTM‐TRS, Fahn‐Tolosa‐Marin Tremor Rating Scale; GPi, globus pallidus internus; Vim, ventral intermediate nucleus of the thalamus.

### Group 1, Extremity Predominant Tremor

In two patients  the main symptom was an asymmetric predominant right postural and intention tremor of the hands with mild dystonic posturing of the hands and mild cervical dystonia (for clinical details see Table 1, patients 1 and 2, Video 1). The disease duration was 23 and 28 years, and both patients reported alcohol responsiveness of the tremor. Both had a negative family history for tremor or dystonia syndromes. According to the current consensus statement, the tremor was classified as dystonic tremor.^10^

**Video 1:** Patient two with asymmetric, predominant postural action tremor of the hands and only mild dystonia of the arms and neck at baseline (segment 1), 3 months post globus pallidus internus‐deep brain stimulation (DBS) (segment 2) and 3 months post ventral intermediate nucleus of the thalamus‐DBS (segment 3).

**FIG. 1:**
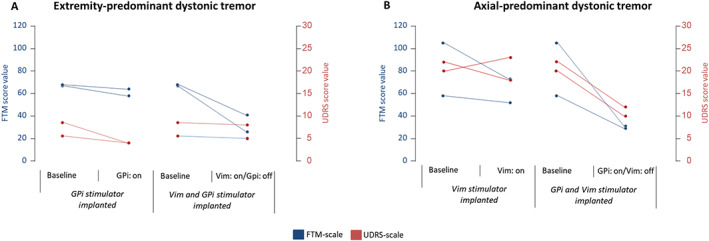
Effect of Vim‐ and GPi‐DBS in four patients with tremor‐dominant cervical dystonia syndromes. Given are FTM‐total‐ (blue) and UDRS‐scores (red) (**A**) Two patients with predominant postural‐action tremor of the arms. (**B**) Two patients with predominant postural‐axial tremor. VIM, ventral intermediate nucleus of the thalamus; GPi, globus pallidus internus; DBS, deep brain stimulation; FTM, Fahn‐Tolosa‐Marin; UDRS, Unified Dystonia Rating Scale.

In a participatory decision‐making process, we decided to target the GPi to achieve a good effect on both symptoms at best. Bilateral GPi stimulation did not significantly decrease the tremor in both patients, but the dystonic symptoms improved by an average of 45% (mean UDRS at baseline: 7 points, at follow‐up with Vim stim OFF/GPi stim ON: 4). As the anti‐tremor effect of GPI‐DBS was not sufficient, additional implantation of Vim‐DBS electrodes on both sides was performed half a year later, from which both patients benefited considerably with a mean tremor reduction of 51% as assessed with the FTM‐TRS total score (mean FTM‐TRS at baseline: 68, with Vim stim ON/GPi stim OFF: 33) (Fig. 1A and Table S1; an exampe of a patient can be viewed at https://onlinelibrary.wiley.com/doi/10.1002/mdc3.13846). Dystonic symptoms were almost unchanged by Vim stimulation.

### Group 2, Axial‐Predominant Tremor

The other two patients had a shorter disease duration (5 and 9 years) with rapid progression of an idiopathic predominant axial postural tremor of the neck and trunk (yes‐yes tremor) with mild cervical dystonia and dystonic hand posturing and no improvement after alcohol intake (Table 1, cases 3 and 4, Video 2). In these patients, bilateral Vim‐DBS implantation was performed first with little effect on the predominant postural axial tremor (24% reduction of the mean FTM‐TRS total score) and no effect on dystonic symptoms. Because of an ongoing severe postural axial tremor, an additional bilateral GPi implantation was performed after 5 and 12 months, resulting in a 63% reduction in FTM‐TRS total score (mean FTM‐TRS at baseline: 81, with Vim stim OFF/GPi stim ON: 30) and a 49% reduction in UDRS score (mean UDRS at baseline: 21, with Vim stim OFF/GPi stim ON: 11). Therefore, both tremor and dystonia were significantly improved by GPi stimulation. Combined Vim/GPi long‐term stimulation showed only little additive effect on tremor (75% mean FTM‐TRS total score reduction against baseline in patients 3 and 4 as compared to 63% GPi‐stimulation alone; mean TRS total score at baseline: 81; with Vim stim ON/GPi stim ON: 21).

**Video 2:** Patient four with rapid progressive, predominant axial tremor of the neck and trunk (yes‐yes) at baseline (segment 1), 4 months post ventral intermediate nucleus of the thalamus‐deep brain stimulation (DBS) (segment 2) and 3 months post globus pallidus internus‐DBS (segment 3).

One patient experienced prolonged wound healing without sequelae after surgical intervention. No other serious adverse events occurred. Side effects of Vim stimulation, such as dysarthria or gait ataxia, occurred after the first electrode implantation with increasing stimulation strength in one patient. These stimulation‐induced side effects did not occur when only the GPi was stimulated.

## Discussion

We are presenting four highly selected patients falling into two different, clinically identifiable tremor phenotypes combining tremor and dystonia, an extremity‐predominant dystonic and an axial‐predominant dystonic tremor. All patients needed DBS‐surgery because of the tremor severity. All four patients did not have a satisfying therapeutic response to the first target implanted. The extremity‐predominant patients received GPi‐DBS first and were found to require Vim‐DBS. The axial‐predominant patients received Vim‐DBS first and were found to require GPi‐stimulation. These four cases are providing new hints for the diagnostic classification of dystonic tremors and for the DBS‐treatment of dystonic extremity and axial tremors.

The diagnosis for our cases may be a matter of debate. According to the current consensus criteria for tremor^10^, ^18^ and for dystonia,^19^, ^20^, ^21^ all four patients were classified as idiopathic tremors with cervical dystonia, although the heterogeneous distribution of tremor (proximal versus distal) and the strong predominance of tremor over dystonic symptoms may suggest pathophysiological heterogeneity. In both patients with predominant arm tremor a dystonic tremor was diagnosed for the following reasons: in both patients, tremor and dystonia affected the same extremity, the arm. Additionally, the tremor was irregular/jerky and had a high degree of right/left asymmetry and patient 2 had a preexisting writer's cramp for several years (initially without tremor). These additional features are in favor of dystonic tremor instead of tremor associated with dystonia or ET plus with dystonic symptoms, which can also present with possible co‐contraction mimicking dystonia.^22^ In the patients with predominant‐axial tremor, classification as dystonic tremor was obvious, as head tremor and cervical dystonia clearly affected the same body region. However, such axial tremor syndromes are very rare and various etiologies must be considered, especially acquired cerebellar pathology and a genetic origin.^23^, ^24^ In our two patients, no acquired (cerebellar) pathology were detectable and genetic testing was not performed. Another difference of axial tremors is that the medical treatment differs from the one of extremity tremors and is less successful.^1^, ^25^, ^26^ Whether the distinction between axial and extremity dominant tremor syndromes will gain differential therapeutic significance will have to be shown in future studies.

The selection of the primary stimulation target may be difficult in cases with severe dystonic tremor syndromes. When tremor is the predominant symptom and only mild dystonia is present, Vim‐DBS is usually recommended as the primary target. If, on the other hand, dystonia is the predominant symptom, GPi‐DBS is recommended as the target.^11^, ^27^, ^28^, ^29^ However, our four cases raise the question of whether other factors, such as the anatomical distribution of the tremor, may help to determine the stimulation target. Such a view is supported by a report of a patient with facial and palatal (midline) tremor because of craniofacial dystonia, where normative connectome analysis showed connections of the GPi with the facial area of the motor cortex, whereas the (failed) Vim did not.^13^ This has also been described in patients with Holmes tremor. In axial and proximal dominant tremor, a positive effect was observed with GPi‐DBS, but not with Vim‐DBS.^30^ Recently, the effect of combined Vim‐ and GPi‐DBS surgery (double target approach) was reported in three patients^29^ (with heterogeneous etiology) and nine patients^31^ (with sporadic tremor dominant dystonia) with marked effects on tremor and dystonia; regarding the effects on tremor, the first three mentioned patients tended to benefit more from GPi‐DBS than from Vim‐DBS. In our cases, the additional stimulation of the second target had only a minor effect or was even not tolerated.

There is no generally accepted optimal target for dystonic tremor despite many short‐^11^, ^32^, ^33^, ^34^, ^35^, ^36^, ^37^, ^38^ and long‐term^27^ case series in the field. There is one recent review of the available cases.^31^ Many of the case reports show a better response of tremor than for dystonia with Vim‐stimulation.^11^, ^38^ Conversely, several case reports found dystonia better improved than tremor following Gpi‐stimulation.^11^, ^39^ Some patients only responded to double stimulation in the GPi and Vim.^11^, ^40^, ^41^ Finally, a number of cases are reported to have the best result in the posterior subthalamic area^33^, ^35^, ^42^, ^43^, ^44^ which is mostly stimulated with the most distal electrode contact(s) of electrodes positioned in the Vim.^17^, ^45^ Our cases fit very well with this experience and propose that two clinically discernible subtypes, the extremity‐predominant and the axial predominant subtype of dystonic tremor may have two different target regions, which lead to the best success. A way to move forward would be to collect the cases in a large registry with a detailed phenotyping that might finally lead to a better understanding of this issue.

From a neurological point of view, the aim is always to minimize the intervention and to avoid a rescue operation, but this will not always be possible. Notably, staged implantation of electrodes into a second target is usually described as well tolerated.^11^, ^29^, ^39^, ^41^ The side effects of stimulation (such as gait ataxia or dysarthria) may be limiting or intolerable in individual cases, as we also witnessed in one of our cases. Therefore, we conclude that staged implantation of four electrodes in the Vim and the GPi can be considered in patients with severe combined syndromes of tremor and dystonia if needed.

Our case series has limitations. First, the number of cases studied is small (four patients). Investigation of the effects in a larger patient population would allow stronger conclusions. Second, no genetic testing was performed in the patients. Therefore, although we can describe the clinical syndrome (axis I) in detail, the etiology (axis II) remains sporadic‐idiopathic.^10^ If a genetic mutation was detected, it would be easier to compare the response to therapy, especially for the rare postural axial tremors. Third, DBS surgery was performed first in the target area where the best possible response was suspected (and not randomly). We cannot completely exclude the possibility that the treatment effects would have been different if an alternative order of target selection had been chosen, although sufficiently long washout periods were chosen before treatment evaluation.

## Conclusion

These cases provide three important lessons: (1) the demonstration of dystonic elements of the clinical presentation does not necessarily allow to predict which stereotactic target (Vim or GPi) will be optimal. (2) Our classification of tremor should mention this, and as clinical classifications should have implications for therapy, this may lead to additions or changes of the tremor classification in the future. (3) If patients with severe tremor do not improve sufficiently with stimulation of one target, a second implantation in the alternative target should be considered.

## Author Roles

(1) Research project: (A) Conception; (B) Organization; (C) Execution. (2) Statistical Analysis: A. Design, B. Execution, C. Review and Critique. (3) Manuscript Preparation: (A) Writing of the First Draft; (B) Review and Critique.

S.P.: 1A, 1B, 1C, 2A, 2B, 2C, 3A, 3B.

J.S.B.: 1A, 1B, 1C, 2A, 2B, 2C, 3A, 3B.

M.A.H.: 1B, 1C, 3B.

K.E.Z.: 1B, 1C, 3B.

A.K.H.: 1A, 1B, 1C, 3B.

D.B.: 1A, 3B.

G.D.: 1A, 1B, 1C, 2A, 2C, 3B.

## Disclosures

**Ethical Compliance Statement**: The study is approved by the Kiel University faculty of medicine ethics committee (D601/20). All patients gave their written consent for the research, video documentation and publication. We confirm that we have read the Journal's position on issues involved in ethical publication and affirm that this work is consistent with those guidelines.

**Funding Sources and Conflicts of Interest:** No specific funding was received for this work. The authors declare that there are no conflicts of interest relevant to this work.

**Financial Disclosures for the Previous 12 Months:** S.P. reports speaker honoraria from Insightec, AbbVie, Medtronic, and Boston Scientific outside the submitted work; travel grants from Desitin and AbbVie; and grant/research funding from Parkinson Fonds Deuschland and UCB Pharma. J.S.B. received research support from Stratmann; speaker's honoraria from Ipsen; and consultant fees from Jazz Pharmaceuticals. M.A.H. reports no disclosures. A.K.H. reports no disclosures. K.E.Z. received research support from an intramural grant from the Christian‐Albrechts University of Kiel and from the Benign Essential Blepharospasm Foundation from Ipsen. She reports speaker's honoraria from Bayer Vital, AbbVie Allergan, and Merz outside the submitted work. She has served as a consultant and received fees from Merz, Ipsen, Stratmann and the German Federal Institute for Drugs and Medical Devices (BfArM). G.D. received fees for lecturing from Boston Scientific; and consulting fees from Boston Scientific, Cavion, Functional modulation. He receives funding for his research to his institution from the German Research Council, SFB 1261, B5, and Medtronic. D.B. received consultancies/advisory board fees for Biogen, BIAL, UCB Pharma, Zambon; honoraria for talks/lectures from AbbVie, Bayser, Biogen, BIAL, UCB Pharma, Zambon, and Desitin; and grants/research funding from Deutsche Forschungsgemeinschaft (DFG), German Parkinson's Disease Association (dPV), BMBF, Parkinson Fonds Deutschland, UCB Pharma, EU, Novartis Pharma, Lundbeck, and the Damp Foundation.

## Supporting information


**TABLE S1.** Effect of additional bilateral Vim DBS surgery after insufficient effect of the bilateral GPi stimulation in patients one and two with predominant postural action tremor of the arms with mild dystonic signs
**TABLE S2.** Effect of additional bilateral GPi‐DBS surgery after insufficient effect of the bilateral Vim stimulation in patients three and four with predominant postural axial tremor
