# Supplementary material for: The Challenge of Choosing the Right Stimulation Target for Dystonic Tremor—A Series of Instructive Cases
Source: Mov Disord Clin Pract. 2023 Aug 22;10(10):1478–84. doi: 10.1002/mdc3.13846 (PMC10585974; doi:10.1002/mdc3.13846)
Supplement: Supplementary file 1 — TABLE S1. Effect of additional bilateral Vim DBS surgery after insufficient effect of the bilateral GPi stimulation in patients one and two with predominant postural action tremor of the arms with mild dystonic signs TABLE S2. Effect of additional bilateral GPi‐DBS surgery after insufficient effect of the bilateral Vim stimulation in patients three and four with predominant postural axial tremor [file MDC3-10-1478-s001.docx]

Suppl. material table 1

| Case 1 | **Baseline** | **GPi on:** | **Vim on / GPi off:** |
| --- | --- | --- | --- |
| FTM-TRS part A | 29 | 20 | 8 |
| FTM-TRS part B | 25 | 23 | 11 |
| FTM-TRS part C | 14 | 16 | 7 |
| FTM-TRS total | 67 | 58 | 26 |
| UDRS | 9 | 4 | 8 |
|  | | | |
| Case 2: |  |  |  |
| FTM-TRS part A | 24 | 20 | 14 |
| FTM-TRS part B | 31 | 28 | 14 |
| FTM-TRS part C | 14 | 16 | 13 |
| FTM-TRS total | 68 | 64 | 41 |
| UDRS | 6 | 4 | 5 |

FTM-TRS = Fahn-Tolosa-Marin Tremor Rating Scale (part A-C and total score), GPi = Globus pallidus internus, off = stimulation off, on = stimulation on, UDRS= Unified Dystonia Rating Scale, Vim = ventral intermediate nucleus of the thalamus.

Suppl. material table 2

| Case 3 | **Baseline** | **Vim on:** | **GPi on, Vim off:** |
| --- | --- | --- | --- |
| FTM-TRS part A | 47 | 25 | 9 |
| FTM-TRS part B | 32 | 29 | 10 |
| FTM-TRS part C | 26 | 19 | 13 |
| FTM-TRS total | 105 | 73 | 31 |
| UDRS | 20 | 23 | 10 |
|  | | | |
| Case 4 |  |  |  |
| FTM-TRS part A | 29 | 21 | 13 |
| FTM-TRS part B | 16 | 15 | 8 |
| FTM-TRS part C | 14 | 17 | 9 |
| FTM-TRS total | 58 | 52 | 29 |
| UDRS | 22 | 18 | 12 |

FTM-TRS = Fahn-Tolosa-Marin Tremor Rating Scale (part A-C and total score), GPi = Globus pallidus internus, off = stimulation off, on = stimulation on, UDRS= Unified Dystonia Rating Scale, Vim = ventral intermediate nucleus of the thalamus.
